# CNS Prophylaxis: How Far Is Routine Practice From the Guidelines? Focus on a Nationwide Survey by the Fondazione Italiana Linfomi (FIL)

**DOI:** 10.3389/fonc.2021.730194

**Published:** 2021-11-05

**Authors:** Guido Gini, Alice Di Rocco, Luca Nassi, Annalisa Arcari, Maria Chiara Tisi, Giacomo Loseto, Attilio Olivieri, Massimo Gentile, Ombretta Annibali, Maria Giuseppina Cabras, Annalisa Chiappella, Chiara Rusconi, Andrés José María Ferreri, Monica Balzarotti

**Affiliations:** ^1^ Clinic of Hematology, Ospedali Riuniti, Ancona, Italy; ^2^ Department of Traslational and Precision Medicine, Sapienza University, Roma, Italy; ^3^ Hematology, Department of Translational Medicine, University of Eastern Piedmont and AOU Maggiore della Carità, Novara, Italy; ^4^ Division of Hematology, Guglielmo da Saliceto Hospital, Piacenza, Italy; ^5^ Department of Hematology and Cell Therapy, San Bortolo Hospital, Vicenza, Italy; ^6^ Division of Hematology, IRCCS Ospedale Oncologico, Bari, Italy; ^7^ Department of Onco-Hematology, Hematology Unit, A.O of Cosenza, Cosenza, Italy; ^8^ Unit of Hematology and Stem Cell Transplantation, University “Campus Bio-Medico”, Rome, Italy; ^9^ Division of Hematology, Ospedale Oncologico Armando Businco, Cagliari, Italy; ^10^ Department of Hematology, Azienda Ospedaliero Universitaria Citta`della Salute e della Scienza di Torino, Torino, Italy; ^11^ Division of Hematology, Ospedale Niguarda Ca’ Granda, Milan, Italy; ^12^ San Raffaele Hospital, IRCCS, Milano, Italy; ^13^ Hematology Unit, IRCCS Humanitas Cancer Center, Milan, Italy

**Keywords:** diffuse large B cell lymphoma (DLBCL), central nervous system involvement, survey, risk assessment, prophylaxis

Central nervous system (CNS) prophylaxis in patients with diffuse large B-cell lymphoma (DLBCL) (1) remains at matter of facts an unmeet clinical need.

In a recent paper, McKay et al. for the British Society of Haematology (BSH) published a good practice in this field (2).

Authors should be commended for the relevant effort to draw management recommendations for routine practice. In brief, they suggested that CNS prophylaxis should be offered to patients with any factor among high (4–6) CNS-IPI, involvement of three or more extranodal sites irrespective of CNS-IPI, involvement of certain extranodal organs (i.e., testes, kidney/adrenal), and in intravascular large B-cell lymphoma; they advise to consider CNS prophylaxis in patients with involvement of breast or uterus. Authors recommend the use of two to three cycles of methotrexate (MTX) at 3 g/m^2^ in infusion over 2–4 hours as early as possible, perhaps intercalating it with R-CHOP therapy, leaving intrathecal chemotherapy only for patients unfit for high-dose MTX. These recommendations resulted from an extensive literature research and followed the BSH guidelines, with the aim to recommend good practice in an area where there is a limited evidence. Literature on CNS prophylaxis in DLBCL (3–10) is mostly based on retrospective studies, where high-risk patients were defined usually by homemade, not validated prognostic factors. Anyway, it is interestingly to understand how much this growing, low-level evidence influenced prophylaxis strategies, and definition and management of high-risk patients in the clinical practice. In other words, it would be important to establish how far routine practice from proposed guidelines and supportive literature is. With this aim, we designed a National survey focused on algorithms used in routine practice to identify DLBCL patients at high CNS risk and on strategies to prevent CNS dissemination in Italian cancer centers. A questionnaire designed after consultation with specialist clinicians and a review of published literature was sent to cancer centers referring to the Fondazione Italiana Linfomi (FIL) in July 2018. The primary objectives of this study were to establish strategies used to detect CNS dissemination and to identify high CNS risk patients and preferred CNS prophylaxis in real-life practice, between 2014 and 2018. More in detail, each center was asked to specify the characteristics of the patients considered at high CNS risk (i.e., CNS-IPI > 4, extranodal sites, biological parameters, and chromosomal abnormalities) as well as the list of extranodal organs considered at high risk of CNS relapse (i.e., paravertebral, kidney, adrenal, massive facial, nasopharynx, testicle, and uterus). Information on procedures routinely used to exclude CNS disease (i.e., type of neuroimaging and/or lumbar puncture) and availability of exams on the cerebrospinal fluid (physico-chemical, cytological, flow cytometric) was requested. Information on the routinely use of genetic and molecular tools like cell of origin, chromosomal translocations, and myc/bcl-2/bcl-6 immunostaining was also collected.

Sixty-three (57%) of the 110 invited FIL centers fulfilled the survey.

The survey suggest a concordance between guidelines and clinical practice regarding the criteria used to define patients with high risk of CNS dissemination: CNS-IPI is considered in 87% of centers, and 71% of centers considered the involvement of extranodal organs with reported risk of CNS dissemination. These findings reflect a diffuse acceptance of CNS-IPI in routine practice, whereas a lower use of high-risk extranodal sites may be due to uncertainties in literature on the prognostic value of some sites.

In fact, 92% of centers considered at high risk for CNS relapse the involvement of at least one of the following extranodal organs: kidney, adrenal gland, nasopharynx, and testicle, which is in line with ESMO guidelines (11). Conversely, consensus is lacking for involvement of breast, orbit, paranasal sinus, and skeleton; probably because assessed series were small, included varied lymphoma entities and were treated with different approaches (12). In agreement with BSH guidelines, only 5% of Italian centers use genetic abnormalities to define CNS risk, which reflects inconsistent results in available literature on double hit lymphoma (13–15) and double expresser lymphoma (16, 17). Although the combined assessment of CNS-IPI and cell of origin by gene expression profiling seems to be associated with a high predictive sensitivity (18), a diffuse use of this strategy to guide CNS prophylaxis indication will require independent confirmatory studies and a wider use of gene-expression profiling in routine practice (19). Patients with double/triple and/or ABC and/or double expresser lymphomas were considered at high risk of CNS relapse in in 38 (60%) centers, whereas only those with double\triple hit lymphoma were considered really high risk in six centers (9%).

CNS disease status was assessed by imaging even among asymptomatic patients in 73% of centers, but only half of them use MRI routinely, the others used the less sensitive whole-brain CT scan. Complete diagnostics in cerebrospinal fluid analysis (CSF; physical-chemical, cytological, flow cytometric) are used in 79% of centers, whereas CSF flow cytometry is not routinely performed in 10 (16%) centers. Meningeal/CSF involvement was defined exclusively by positive CSF cytology examination in 19 (30%) centers, whereas the only positive flow cytometry was enough to define this event in 41 (65%) centers. Noteworthy, this is not discussed on international guidelines, but a large retrospective study suggests that most patients with positive CSF flow cytometry and negative CSF cytology will develop more evident CNS disease early (20).

There is gap between guidelines and routine practice regarding the type of CNS prophylaxis; only 40% of participating centers use intravenous MTX at a dose of 1.5 to 3 gr/sqm), with or without intrathecal drug delivery, as CNS prophylaxis, whereas 58% of centers use only intrathecal chemotherapy. This may reflect discrepancies between previously published guidelines: NCCN suggests equally intrathecal and intravenous chemotherapy (21), while ESMO guidelines recognize that intrathecal injections of methotrexate may not be an optimal method, and that intravenous high-dose methotrexate is associated with a lower CNS recurrence rate (11).

This survey shows real-life practice in a representative group of Italian hematological centers. However, it exhibits a few limitations. In particular, prevalently large- or medium-size centers participated, which could have introduced an interpretation bias, mostly in the case small center could show a lower adherence to international guidelines. This survey was produced in Italy and compared with recommendations written by UK colleagues, with consequent weakness regarding differences in patient managements, reimbursement of therapies, among others. With these limitations, this survey highlights the substantial inter-center differences in the use of methods of diagnosis and prophylaxis of CNS involvement in DLBCL patients. The growing literature has been progressively incorporated in international guidelines, but level of evidence of available studies should be improved to draw undebatable recommendations. These achievements should be followed by educational efforts to disseminate these recommendations so that they will soon be incorporated into routine practice.

## Data Availability Statement

The original contributions presented in the study are included in the article/supplementary material. Further inquiries can be directed to the corresponding authors.

## Author Contributions

GG, AR, MS, LN, AA, MT, GL, MG, OA, AF, and MB contributed to conception and design of the study. All authors contributed to manuscript revision, read, and approved the submitted version.

## Conflict of Interest

The authors declare that the research was conducted in the absence of any commercial or financial relationships that could be construed as a potential conflict of interest.

## Publisher’s Note

All claims expressed in this article are solely those of the authors and do not necessarily represent those of their affiliated organizations, or those of the publisher, the editors and the reviewers. Any product that may be evaluated in this article, or claim that may be made by its manufacturer, is not guaranteed or endorsed by the publisher.
